# 

**Published:** 1891-10

**Authors:** 


					﻿VOL XI.
OCTOBER, 1891.
NO. 10.
THE
HOMEOPATHIC pHY£lClA]\I,
A Monthly Journal of Homoeopathic Materia Medica
and Clinical Medicine.
"He is a freeman whom truth makes free, and all are slaves beside,"
"Seek the Truth: come whence it may, cost what it will.”
EDITED AND PUBLISHED BY
WALTER M. JAMES, ME. D.
PHILADELPHIA :
No. 1125 SPJRTTC3E STREET.
LONDON
ALFRED HEATH & CO., Sole Agents fob Great Britain,
114 Ebury Street, Eaton Square.
43?* Yearly Subscription, $2.50, invariably in advance. Single numbers, 95 cts.
Entered at the Philadelphia Poet-Office as Second-Claee Mall Matter.
				

